# Host-microbe interactions at the blood-brain barrier through the lens of induced pluripotent stem cell-derived brain-like endothelial cells

**DOI:** 10.1128/mbio.02862-23

**Published:** 2024-01-09

**Authors:** Nadine Vollmuth, Jon Sin, Brandon J. Kim

**Affiliations:** 1 Department of Biological Sciences, University of Alabama, Tuscaloosa, Alabama, USA; 2 Department of Microbiology, Heersink School of Medicine, University of Alabama at Birmingham, Birmingham, Alabama, USA; 3 Center for Convergent Biosciences and Medicine, University of Alabama, Tuscaloosa, Alabama, USA; 4 Alabama Life Research Institute, University of Alabama, Tuscaloosa, Alabama, USA; Ohio State University, Columbus, Ohio, USA; Indiana University, Indianapolis, Indiana, USA

**Keywords:** stem cell, meningitis, meningoencephalitis, host-pathogen interactions, blood-brain barrier

## Abstract

Microbe-induced meningoencephalitis/meningitis is a life-threatening infection of the central nervous system (CNS) that occurs when pathogens are able to cross the blood-brain barrier (BBB) and gain access to the CNS. The BBB consists of highly specialized brain endothelial cells that exhibit specific properties to allow tight regulation of CNS homeostasis and prevent pathogen crossing. However, during meningoencephalitis/meningitis, the BBB fails to protect the CNS. Modeling the BBB remains a challenge due to the specialized characteristics of these cells. In this review, we cover the induced pluripotent stem cell-derived, brain-like endothelial cell model during host-pathogen interaction, highlighting the strengths and recent work on various pathogens known to interact with the BBB. As stem cell technologies are becoming more prominent, the stem cell-derived, brain-like endothelial cell model has been able to reveal new insights *in vitro,* which remain challenging with other *in vitro* cell-based models consisting of primary human brain endothelial cells and immortalized human brain endothelial cell lines.

## Blood-brain barrier model

Microbe-induced meningoencephalitis/meningitis is a potentially fatal infection of the central nervous system (CNS) that occurs when pathogens are able to interact with and cross the blood-brain barrier (BBB) and induce inflammation. Infectious meningoencephalitis/meningitis can be caused by a number of fungal, viral, or bacterial pathogens, with viruses and bacteria being the most common culprits (1–3). Known viral agents that cause meningoencephalitis include herpes simplex virus (4, 5), human herpesviruses 6 and 7 (6, 7), enteroviruses (8), Japanese encephalitis virus (9, 10), West Nile virus (11, 12), cytomegalovirus (13, 14), varicella zoster virus (15, 16), Epstein-Barr virus (17, 18), human immunodeficiency virus (19, 20), and rabies virus (21–23). Bacteria such as *Escherichia coli* K1 (24), *Mycobacterium tuberculosis* (25), *Listeria monocytogenes* (26), *Streptococcus pneumoniae* (27), *Haemophilus influenzae* type b (28), Group B *Streptococcus* (GBS) (29, 30), and *Neisseria meningitidis* (Nm) (31) can cause bacterial meningitis in children and adults (3). Many of these pathogens, namely *E. coli* K1, *S. pneumoniae*, *H. influenzae*, Group B *Streptococcus,* and *N. meningitidis,* are persistent colonizers of mucosal surfaces in humans and, under certain conditions, can enter the circulation and interact with brain barriers to gain access to the CNS (3). Despite modern medical intervention, bacterial meningitis is still a fatal disease worldwide and can even leave survivors with permanent neurological sequelae (32, 33). Modeling host-pathogen interactions at the BBB remains challenging *in vitro* due to the complex nature of the BBB; however, recent advances in stem cell technologies offer advantages to more traditional cell-based models and could provide insight on novel pathogen-BBB interactions.

**Fig 1:**
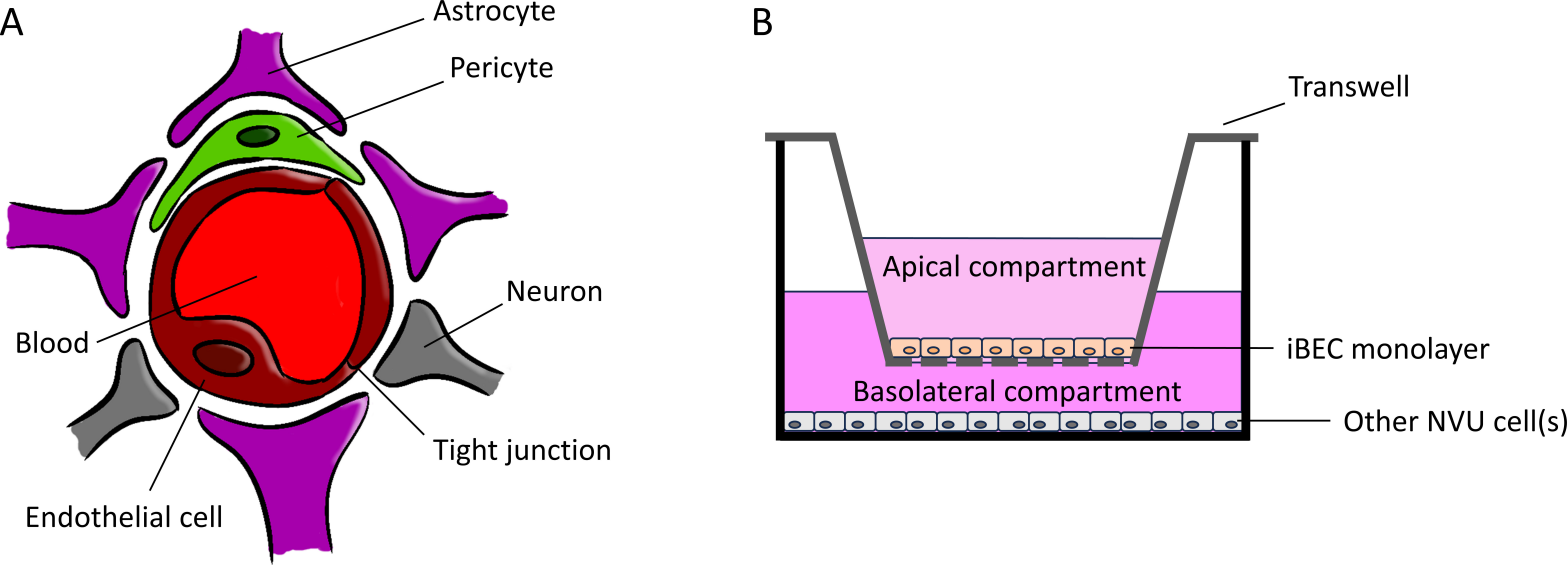
Scheme of the blood-brain barrier and the possibilities of how to use the induced brain microvascular endothelial cells (iBECs). (**A**) Scheme of the neurovascular unit (NVU). BECs comprise the blood-brain barrier, along with pericytes, astrocytes, and neurons forming the NVU. The cells of the NVU contribute to the tight barrier properties of BECs, which enable the maintenance of central nervous system homeostasis by the creation of a highly selective barrier. (**B**) Scheme of the iBEC model usage. In addition to cultivating an iBEC monolayer on a tissue culture plate, iBECs can also be utilized in a transwell system. This setup allows for measurement of barrier integrity and provides an apical and basolateral compartment. Furthermore, the transwell system enables the cultivation of iBEC monolayers with other cell types, including all cell types of the NVU for observation of the interplay between them.

The BBB is formed of brain microvascular endothelial cells (BECs) and is a component of the neurovascular unit (NVU), which additionally comprises pericytes, astrocytes, and neurons (34–36). Working in concert, the cells of the NVU contribute to the tight barrier properties of the BECs, and this allows the NVU to maintain CNS homeostasis by creating a highly selective barrier (37–39) (Fig. 1A). BECs not only have nutrient transporters and multidrug efflux transporters to maintain homeostasis and tightly control entry of molecules into the CNS, but they also exhibit a low endocytosis rate and complex intracellular tight junctions to separate the circulation of the brain (34, 35, 40–45), thus, transcellular and paracellular flux are greatly limited (46). Pathogens can block these efflux transporters, destroy the tight junctions, and increase the rate of endocytosis to overthrow the key aspects of the BBB, but in many infections, it remains poorly understood how the pathogens are able to cross the BBB. Possible mechanisms that pathogens utilize to cross the barrier are transcellular traversal, paracellular traversal, or the Trojan horse mechanism. While the pathogens penetrate between the barrier cells either with or without evidence of tight junction disruptions at the paracellular transversal, the transcellular transversal is through the cells without tight junction disruption and no detection of pathogens between the barrier cells. The Trojan horse mechanism involves infected phagocytes being used to transmigrate through the barrier cells. Therefore, it is important to have good models that reflect as many of the key aspects of the BBB as possible (47–49). *In vitro* cell-based studies traditionally utilized immortalized human BEC cell lines and primary human BECs (50–55), which have served as valuable tools to begin identifying host-pathogen interactions. However, many of these *in vitro* models rapidly lose native BBB properties when removed from the brain microenvironment (45, 56). Additionally, issues may arise due to limited postmortem tissue availability or model cells being derived from patients with a predisposing disease such as epilepsy, which can inherently have an altered BBB phenotype (46, 56, 57). Stem cell technologies have recently offered a renewable source for BECs and have added advantages of being highly scalable and possessing many BBB phenotypes (45, 58–69).

Human pluripotent stem cells can be either human embryonic stem cells obtained from the inner cell mass of human blastocytes (70) or induced pluripotent stem cells (iPSC) generated from somatic cells that have been reprogrammed to become pluripotent. iPSCs have the potential to differentiate into any cell type of the body, which includes brain-like endothelial cells (45, 71–73). BECs derived from iPSC sources (iBECs) show many expected characteristics of BECs *in vitro*. Since their inception, differentiation protocols have continued to evolve to better recapitulate *in vivo* BEC properties such as high transendothelial electrical resistance (TEER), transporters [P-glycoprotein (P-gp), GLUT1], efflux transporter activity, and essential BBB junctional proteins (Claudin-5, ZO-1, Occludin, VE-Cadherin), and other factors such as PECAM-1, VEGFR2, and von Willebrand factor (58, 74–81). iBECs display superb organization of tight junctions, express functional nutrient and efflux transporters, and the permeability of pharmacological agents correlates with that seen *in vivo* (59, 82–85) (Table 1). Further improvements have increased endothelial identity through the addition of small molecules or erythroblast transformation-specific factors (60, 86–89). Additionally, recent work has demonstrated that iBECs in co-culture with other cell types of the NVU greatly improve modeling and can be derived in an isogeneic fashion to construct a NVU model from a single stem-cell source (81, 90, 91) (Fig. 1B).

**TABLE 1:** Comparison of the BBB and different models to study the BBB

	Transporter	TEER (Ohm × cm^2^)	Junctions	Endothelial marker	Reference
*In vivo* BBB	P-gp, BCRP, Mrp-1	>1,000	Occludin, ZO-1, Claudin 5, JAM-A	PECAM-1, VE-cadherin, von Willebrand factor	(92)
iBECs	P-gp, BCRP, Mrp-1,2,4 and 5, Glut-1	250–6,000	Occludin, ZO-1, Claudin 5	PECAM-1, VE-cadherin, von Willebrand factor	(59, 60)
hCMEC/D3 (immortalized human endothelial cell lines)	P-gp, BCRP, Mrp-1	40–200	Occludin, ZO-1, Claudin 1,3,5, and 12, JAM-A	PECAM-1, VE-cadherin, von Willebrand factor	(51, 93, 94)
HBMEC (immortalized human endothelial cell lines)	P-gp, Glut-1	20–350	Occludin, ZO-1, Claudin 1 and 3, weak Claudin 5, JAM-1	VE-cadherin, von Willebrand factor	(94–96)
Primary human endothelial cells	P-gp, Glut-1	20–200	Occludin, ZO-1 and 2, Claudin 1, 3, and 5, JAM-2	PECAM-1, von Willebrand factor	(50, 97)

The iBEC model has been shown to possess great utility in studying infectious diseases caused by neurotropic pathogens and has been demonstrated to be a particularly valuable tool for interrogating human-specific pathogens. Furthermore, studies investigating relationships between genetic backgrounds and altered BBB phenotypes have been conducted by deriving iBECs from iPSCs carrying single nucleotide polymorphisms of interest (58, 67, 81, 98, 99). To date, the iBEC model has proven useful to study a variety of CNS disorders and has recently been introduced into the infectious disease community. It has demonstrated utility with various pathogens and has opened the doors to further elucidate the complex host-pathogen interactions at the BBB. In the following review, we will examine the different pathogens the iBEC model has been used thus far to reproduce findings from other models of host-pathogen interactions. Additionally, we will examine novel findings that could not be explored in other *in vitro* models to date.

### Fungi

Most fungal infections are restricted to mucosal environments; however, some like *Aspergillus* spp. (100), *Coccidioides immitis* (101), *Candida albicans* (102), *Cryptococcus neoformans* (103), *Histoplasma capsulatum* (104), or *Paracoccidiodes brasiliensis* (105) can migrate to the CNS and induce a life-threatening encephalitis. To reach the CNS, fungi must cross the BBB, but mechanisms of entry are not fully understood. Most *Aspergillus* strains involved in aspergillosis produce a mycotoxin, gliotoxin, which was found in patient sera at high levels with amounts up to 785 ng/mL (106). Therefore, it was investigated whether a chemical interaction through the secretion of the gliotoxin would be involved in the interaction between BBB and *Aspergillus*. Using the iBEC model and the purified gliotoxin from *Gliocladium fimbriatum*, it was discovered that intoxicated iBECs reduced TEER and increased BBB permeability. Impairment of cell matrix interactions was found to cause barrier disruption rather than tight junction changes, revealing a previously unknown mechanism that might be used by *Aspergillus fumigatus*. The gliotoxin had low BBB permeability, suggesting that it might in fact interact with membrane proteins, causing alterations in F-actin distribution (107). Mycotoxins seem to play an important role in the interaction between toxin-secreting fungi and the BBB, which could also be tested for other encephalitis-causing fungi using the iBEC model. Future work should determine whether a gliotoxin-deficient *Aspergillus* could disrupt the BBB compared with the wild type (WT).

### Viruses

**Fig 2:**
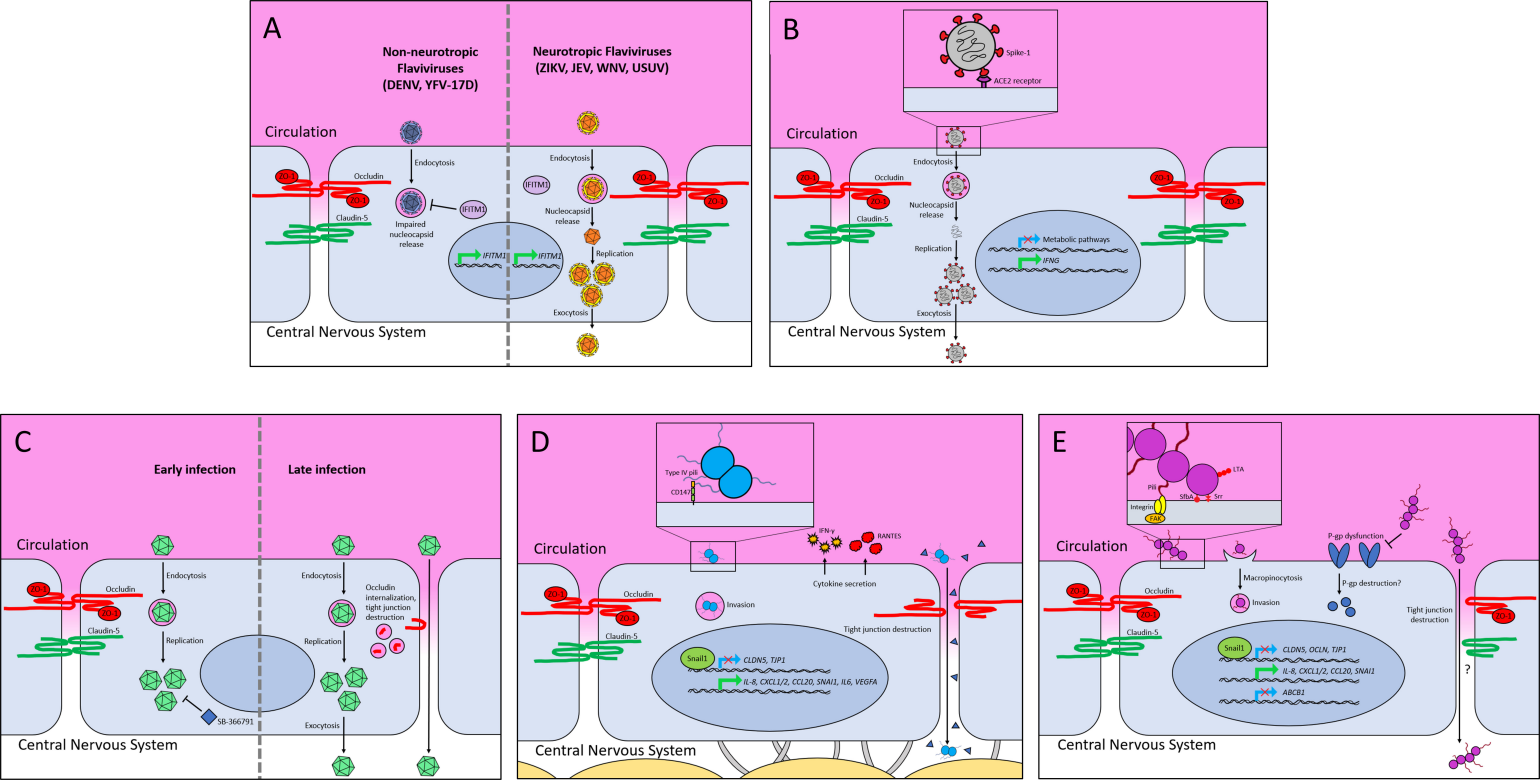
Cartoon of neurotropic viruses and bacterial pathogens interacting with the blood-brain barrier. (**A**) Non-neurotropic (left side) and neurotropic flaviviruses (right side) infection of iBECs. Brain endothelial cells produce IFITM1 (interferon-induced transmembrane proteins), which protects from non-neurotropic flaviviruses such as dengue virus (DENV) and yellow fever virus-17D (YFV-17D) by impairing nucleocapsid release. Neurotropic flaviviruses such as Japanese encephalitis virus (JEV), Zika virus (ZIKV), West Nile virus (WNV), and Usutu virus (USUV) can resist the antiviral impacts of IFITM1 and replicate in the brain endothelial cells. (**B**) SARS-CoV2 accessing the central nervous system. Spike-1 protein interacts with the ACE2 (angiotensin-converting enzyme 2) receptor before being endocytosed into brain endothelial cells. SARS-CoV2 induces *IFNG* (interferon-y) expression and represses genes for the metabolic pathways in iBECs. The newly produced virions get released without disturbing tight junction integrity. (**C**) Coxsackievirus B3 (CVB3) interaction with brain endothelial cells at early (left side) and late (right side) infection. During early infection, CVB3 gets endocytosed, replicates in iBECs, and reaches the brain side of the endothelial cells. Treating the cells with the transient receptor potential cation channel subfamily V member 1 inhibitor SB-366791 reduces CVB3 infection by interfering with viral replication. During late infection, Occludin is internalized and tight junction destruction is observed. (**D**) *Neisseria meningitidis* (Nm) infecting iBECs. Adherence and invasion are initiated through binding with its type IV pili, the cell receptor CD147, and interacting with integrins. Nm infection leads to cytokine secretion (IFN-y and RANTES) and tight junction destruction. Additionally, Nm activates the expression of *IL-6* (interleukin-6), *IL-8* (interleukin-8), *CXCL1/2* [chemokine (C-X-C motif) ligand 1/2], and *SNAI1* (snail family transcriptional repressor 1). Snail-1, the tight junction repressor, inhibits the expression of the tight junction genes *CLDN5* (claudin 5) and *TJP* (tight junction protein 1). Additionally, gene expression of *VEGFA* (vascular endothelial growth factor A) is upregulated. Through tight junction disruption, a paracellular route to the brain-side of iBECs is observed by Nm transit and permeability to NaF (triangles). (**E**) Group B *Streptococcus* (GBS) infecting iBECs. Adherence is promoted by the adhesins PilA (pilus), SfbA (fibronectin-binding protein), and Srr (serine-riche repeat) glycoprotein. IagA glycosyltransferase is predicted to be the cell membrane anchor for lipoteichoic acid (LTA) and plays an important role in BBB invasion by GBS. The pilus tip PilA binds collagen and initiates the interaction with the integrins on the endothelial cell surface. Altogether, GBS initiates bacterial uptake and an upregulation of proinflammatory chemokines and cytokines [*IL-8*, *CXCL1/2* (chemokine (C-X-C motif) ligand 1/2), *CCL20* (chemokine (C-C motif) ligand 20)]. Macropinocytosis contributes to GBS invasion of iBECs. Additionally, GBS infection leads to tight junction destruction through the upregulation of *SNAI1* (snail family transcriptional repressor 1), a tight junction repressor, decreasing tight junction proteins such as Claudin-5, Occludin, and ZO-1 by repressing gene transcription of *CLDN5* (Claudin-5), *OCLN* (Occludin), and *TJP1* (tight junction protein 1). With the loss of tight junctions, a paracellular route may be used additionally by GBS. Finally, the P-gp efflux transporter is functionally inhibited during GBS infection, and expression of the P-gp gene *ABCB1* is repressed. There may be a destruction of P-gp, which leads to the loss of its function.

Zika virus is neurotropic, suggesting that the BBB might be permissive to the virus (108, 109). Zika virus generally causes mild infections in adults, but cases of microcephaly, Guillain-Barre syndrome, and other neurological conditions connected with Zika virus are increasing (108, 110). In iBECs, Zika virus does not compromise BBB integrity, which is evidenced by unaltered TEER suggesting transcytosis may be the primary mode of crossing the barrier (109). Some studies in primary human BECs also observed that Zika virus infects these cells, triggers activation of inflammatory cytokines, and gets released basolaterally without overt barrier disruption (111, 112), but other reports suggest that Zika virus may cross the BEC barrier through paracellular transit or through the Trojan horse strategy inside leukocytes to gain access to the CNS in primary human BECs and immortalized BEC cell lines (113–116). Further investigation is needed to determine how Zika virus crosses the BBB, but slight disruption of the barrier might be induced by the virus at later time points, even in the studies that do not observe higher permeability (109, 112). BECs could also be a reservoir for Zika virus enabling persistent infection (109, 111) (Fig. 2A).

The utility of the iBEC model in the context of neurotropic viral infection was highlighted in a recent paper, which found that iBECs naturally express antiviral interferon-induced transmembrane proteins (IFITMs) that specifically protect BECs from flaviviruses, such as dengue virus and yellow fever virus-17D. This differentiation between neuroinvasive and non-neuroinvasive viruses could not be observed in the hCMEC/D3 cell line (117). Comparisons with other barrier-forming cells revealed that, in addition to BECs, the blood-testes barrier and the retinal pigmented epithelium also express high levels of IFITMs, demonstrating a mechanism for viral exclusion from privileged sites (117). Neurotropic flaviviruses such as Japanese encephalitis virus, Zika virus, West Nile virus, and Usutu virus, however, can circumvent the antiviral impacts of IFITM1 and still impact barrier-forming cells such as iBECs (117). Whereas non-neuroinvasive viruses were inefficient in infecting the iBEC model. Importantly, utilization of the iBEC model in this context was instrumental for the discovery of novel antiviral factors in barrier-forming cells (117) (Fig. 2A).

In 2020, the SARS-CoV2 pandemic struck the world, with many patients experiencing neurological symptoms, suggesting that SARS-CoV2 may be neurotropic. While SARS-CoV2 primarily affects the respiratory system, systemic damage was observed also in other organs such as the kidney, heart, liver, and brain (118, 119). Therefore, the susceptibility of the BBB is of interest in determining the potential entry of SARS-CoV2 into the CNS. The primary surface receptor for SARS-CoV2, ACE2, is expressed in endothelial cells, suggesting that BECs may be susceptible to infection (120–122). iBECs were shown to express ACE2 at levels comparable to human brain tissue (119). Whereas the hCMEC/D3 cell line could not be infected with SARS-CoV2 due to the ectopic ACE2 expression required to infect the iBEC model (119, 123). Upon infection with SARS-CoV2, iBECs responded with a profound transcriptional response, including downregulation of metabolic processes and upregulation of interferon-gamma-mediated signaling pathways. These findings were then confirmed in postmortem COVID-19 patient tissue. Autopsy samples of COVID-19 patients and biopsy samples as controls were taken for spatial transcriptomic analysis using the Nanostring Digital Spacial Profiler platform. The specific regions of interest were those surrounding cortical vessels for this approach. Infected tissue showed an increase in *IFITM1* and *IFITM2* expression, which was also seen in the iBEC model by mRNA sequencing and qRT-PCR. Furthermore, biological themes, which have been upregulated in patient samples, were significantly enriched and downregulated themes were echoed in the iBEC model (119). Genes of the interferon-gamma receptor complex components have been found to be upregulated in immortalized BECs (124). Studies investigating SARS-CoV2 traversal across the BBB revealed that the Spike 1 protein of SARS-CoV2 crosses the BBB in mice but was not transported in iBECs, which could mean that this is not the case in humans or that active infection of BECs is required for CNS transit (125) (Fig. 2B).

Non-polio enteroviruses, such as echoviruses and coxsackieviruses, are a leading cause of aseptic meningitis, especially in very young children. Coxsackievirus B3 (CVB3) infections are generally subclinical or cause a mild flu-like illness; however, sometimes they can provoke myocarditis (126) and pancreatitis (127). Importantly, CVB3 is also neurotropic and one of the leading causes of non-bacterial, aseptic meningoencephalitis (128, 129). Despite this, mechanisms of viral access to the CNS remain unclear (130, 131). Recently, iBECs have been utilized to study CVB3 interactions with BECs. iBECs were shown to be susceptible to CVB3 infection, although interestingly, they could harbor infection for long periods and remain resistant to CVB3-mediated cell death. In contrast, CVB3-permissive cells such as HeLa cells rapidly display cytopathic effect and cell death following infection. CVB3 was also shown to disrupt iBEC tight junctions, and TEER was reduced at late time points suggesting BBB disruption. Surprisingly, however, even with the observed barrier dysfunction and continued viral shedding, the iBEC monolayer remained intact, supporting the notion that iBECs may be susceptible to persistent CVB3 infections. Finally, it was observed that inhibiting known pro-viral pathways resulted in a reduction in CVB3 abundance and replication (130). In HeLa cells, CVB3 was reported to rely on transient receptor potential cation channel subfamily V member 1 (TRPV1)-mediated mitochondrial fragmentation (132). This was evidenced by the observation that the TRPV1 inhibitor SB-366791 significantly reduced CVB3 infection. Similarly, treating iBECs with SB-366791 also limited CVB3 infection, suggesting that the iBEC models may be useful for testing potential antiviral compounds at the BBB (130, 132) (Fig. 2C).

### Bacteria

In addition to neurotropic viral infections, the iBEC model has been used to study two clinically relevant bacterial meningeal pathogens: Nm and *Streptococcus agalactiae* (Group B *Streptococcus*, GBS) (133–139).

Nm, a human-exclusive pathogen, asymptomatically colonizes the upper respiratory tract; however, it is the leading cause of purpura fulminans and meningitis in susceptible individuals (3, 140, 141). While a vaccine has been developed, protection is only conferred on inoculated individuals; thus, Nm poses a high health risk in underdeveloped countries and sub-Saharan Africa (140, 142–144). Due to Nm being an obligate human pathogen, the establishment of suitable animal models to study the mechanisms by which the bacteria colonize and traverse host barriers remains challenging (145). In the iBEC model, it was shown that Nm disrupts tight junctions and upregulates *SNAI1* (Snail-1, a transcription repressor of tight junction components) (134, 138, 146). In immortalized BECs, Occludin cleavage could be observed upon Nm infection, contributing to the loss of tight junction integrity (147). The iBECs showed a decrease in protein levels for ZO-1, Claudin-5, and Occludin at later time points, and additionally for Occludin, a second band at lower molecular weight, suggesting a cleavage product as seen in the immortalized BECs, was observed. Another upregulated factor upon infection is vascular endothelial growth factor A. The upregulation seen upon RNA-Seq was confirmed using qPCR, which was specifically interesting because it had been previously implicated to be a target of *E. coli* for disrupting the BBB and contributes in general to BBB disruption (134, 148, 149). Furthermore, Nm increases proinflammatory cytokines in iBECs, similar to previous observations in immortalized BEC cell lines (134, 150, 151). Interestingly, the upregulation of RANTES (a chemokine secreted by platelets) and IFN-γ could be detected using the model, which is commonly found in the cerebrospinal fluid of patients suffering from bacterial meningitis caused by different pathogens (134, 152, 153). Microarray analysis of an immortalized BEC cell line infected with Nm revealed similar regulations as the RNA-Seq analysis, such as upregulation of RANTES and IL-6, but in the microarray, the regulation of IFN- γ was not detected (154). Moreover, iBECs express CD147, a pilus receptor localizing with meningococcal colonies (134). A study with immortalized BEC cell lines revealed that CD147 is a type IV pilus receptor (155). Other important BEC receptor Nm invasion recruits are the fibroblast growth factor receptor 1-IIIc (FGFR1-IIIc), the laminin receptor, and the tyrosine kinase receptor ErbB2, which could be investigated in the iBECs (156–160). A major strength of the iBEC model is that they respond to other CNS cell types in a co-culture system (59, 60, 90, 91). Using iBECs in a co-culture system with leptomeningeal cells leads to consistent results observed with monoculture for Nm despite the increased barrier function as measured using TEER due to the co-culture (135). These findings may suggest a potential paracellular route for Nm transversal of brain barriers; however, further studies are required (Fig. 2D).

GBS is the leading cause of neonatal meningitis and an emerging pathogen in specific adult populations. Despite modern advances in medical intervention, GBS remains a significant cause of death in neonates. Even surviving infants often suffer serious long-term neurological sequelae (161–163). Previously, it had been discovered that GBS infection leads to cytokine and chemokine upregulation responsible for neutrophil recruitment in immortalized BEC cell lines (164). The same response could be observed in the iBEC model, mimicking those found *in vivo* (138). Using iBECs, it was discovered that GBS inhibits the P-gp transporter, a key BBB efflux transporter, although its impact on infection is unknown. This was first observed in iBECs and then affirmed in a P-gp overexpressing cell line and a GBS infection mouse model. The *in vivo* study showed less co-localization between P-gp immunolabeling and endothelial cells in GBS-infected mice (139). Additionally, it was discovered that GBS may activate macropinocytosis (typically tightly regulated at the BBB) to invade BECs, which was corroborated in immortalized BEC cell lines (53, 137). However, a key mechanism of GBS-mediated barrier dysfunction is the upregulation of the tight junction repressor Snail-1, which was recapitulated in the iBEC model. GBS-induced Snail-1 expression was observed in immortalized BEC cell lines and murine and zebrafish models, leading to a decrease in the tight junction components Occludin, Claudin-5, and ZO-1. GBS-infected mice showed a significant increase in *Snai1* transcripts compared to uninfected mice. Furthermore, it could be observed that Snail-1 co-localized with von Willebrand factor in mice brain tissue, supporting Snail-1 expression in BECs during active infection. Claudin-5 and Occludin expression of endothelial cells isolated from infected mice were significantly lower than that from uninfected mice. In zebrafish, the *Snai1* homolog *snai1a* was upregulated upon GBS infection, and its knockdown resulted in attenuated GBS passage to the brain. Moreover, *Snai1a* inhibition lowered GBS-induced mortality in zebrafish larvae (138, 146). Tight junction proteins Occludin, Claudin-5, and ZO-1 are decreased during GBS infection, and this coincides with a loss of TEER in the iBEC model (138). Finally, iBECs express similar cellular receptors necessary for GBS attachment, such as the β1 integrin (165, 166). Thus, unsurprisingly, bacterial mutants with impaired virulence factors display reduced attachment and invasion on iBECs (3, 138, 167–174). The invasion-associated gene (*iagA*) has a specific function in promoting host uptake of GBS, and mutations of this gene decrease invasion. IagA glycosyltransferase is predicted to be the cell membrane anchor for lipoteichoic acid (LTA). Furthermore, Glucose(β1–6)Glucose(β1–3)(gentiobiosyl)diacylglycerol synthase is encoded by *iagA* leading to impaired LTA cell membrane anchoring (174). For adherence to the brain endothelium, GBS has the pilus protein PilA, which binds collagen, an extracellular matrix component, and enhances attachment and uptake (167). Furthermore, the collagen PilA complex engages β1 integrins to promote not only attachment but also proinflammatory chemokine release (165). Streptococcal fibronectin-binding factor A (SfbA) contributes to GBS invasion of the BEC and bloodstream survival (166, 171). The serine-riche repeat (Srr) glycoprotein binds fibrinogen through a dock, lock, and latch mechanism, and contributes to the BEC invasion, but only Srr2 is associated with hypervirulence (168, 170, 173). All of these GBS virulence factors were attenuated in bacterial-BEC interactions using the iBEC model and have been previously elucidated in immortalized BEC cell lines. *In vivo*, *iagA* and *pilA* mutants showed a lower bacterial load in the brains of mice and a lower mortality rate compared to mice infected with WT GBS (165, 174). A lower bacterial load compared to the WT GBS in mice could also be observed for the *srr2* mutant (169). Interestingly, SfbA showed a role for both attachment and invasion in the iBECs, which was not seen in the immortalized BEC cell line (138). In *in vivo* studies, the *sfbA* mutant showed lower bacterial load in the brains of mice compared to WT GBS, but additionally, recovery from the blood of the mice was also reduced for the mutant (166). These findings on the previously known virulence factors iagA, PilA, SfbA, and Srr2 suggest that the iBEC model recapitulates the results of earlier studies using immortalized BEC cell lines and *in vivo* models. Moving forward, researchers could begin to probe phenotypes of the BBB, which may be limited in immortalized BEC cell lines, and discover novel bacterial virulence factors impacting these BBB characteristics directly (Fig. 2E).

### Future

iBECs are reliable and readily scalable similar to immortalized cell lines, but they maintain BBB-like phenotypes, increasing their potential to uncover novel host-pathogen interactions. In addition to showing physiologically relevant properties, these cells offer the prospect of studying host-pathogen interactions at the BBB that are not readily modeled in other systems (138). Moreover, the use of human-derived iBECs allows for the study of obligate human pathogens, such as Nm (134, 135). Furthermore, the model could be used in pandemic preparedness, as emerging threats are bound to resurface and the iBECs could depict postmortem tissue findings. Therefore, iBECs can be used not only to gain a better insight into the mechanism but also to test treatments if the threat has a neurotropic component. Moreover, the possibility of generating iBEC models with different genetic backgrounds could reveal if certain groups have a higher chance of infection or are resistant. iBECs can also be incorporated into more complex systems, such as microfluidic organ-chip-based models (58) and have proven useful in examining other CNS disorders (175–180).

Though iBECs are rapidly emerging as a useful model in the field of BBB biology, improvements are still ongoing. The model has some limitations because the iBECs express not only endothelial markers but also contain a mixed phenotype with epithelial cells. Therefore, differentiation needs to be confirmed by checking for the presence of the requisite endothelial markers and measuring barrier formation. In addition, epithelial cells also generally have a high TEER. Another characteristic that needs to be evaluated for the iBEC model is its complete immunophenotype. Furthermore, the model should be used for particular applications if it is suitable, and as for any other model, the results should be validated in complementary models or/and *in vivo* (87, 88, 181). Many studies using the iBEC model utilize fibronectin and collagen type IV as an adhesion layer for the iBECs, but fibronectin plays a major role during development and is not anymore in healthy adults. The basement membrane *in vivo* separating endothelial cells from connective tissue consists of laminin and collagen type IV. A study found that using fibronectin and collagen IV exhibited an activated endothelial phenotype, while laminin 511-E8 exhibited a resting endothelial phenotype. Furthermore, laminin 511-E8 improved the barrier properties of the iBECs through an improved junctional protein expression, reduced stress fibers, a more sustained TEER stability, and enhanced physiological responses to shear stress. Isolation and purification of specific isoforms of native pure laminins from tissue is difficult, which limits the use of tissue-specific laminins for *in vitro* studies. The production of recombinant laminins and fragments only recently started and could prove cost prohibitive (182–184). Besides co-cultures with other NVU cell types, viral transfection and small molecule treatments are currently being explored to improve endothelial cell identity (86–88, 185). One such small molecule treatment, cARLA, provides an affordable and easy-to-use method that robustly raises BBB properties in a range of models (86).

In summary, the iBEC model provides the ability to address host-pathogen interactions on an improved system that could be utilized with a variety of pathogens. Ongoing and future work using iBECs during infectious disease could identify novel interactions impacting BBB function directly; thus, this model presents immense potential for identifying key therapeutic targets to limit CNS infection and dysfunction.
